# Comprehensive Analysis of Small RNA Modifications in *Arabidopsis thaliana* and Their Dynamics During Seed Germination

**DOI:** 10.3390/metabo15050319

**Published:** 2025-05-10

**Authors:** Liu-Cheng Jiang, Meng Men, Xuan-Jun Cui, Ren-Jie Zeng, Shu-Yi Gu, Tian Feng, Chen Zeng, Tiantian Ye, Jun Xiong, Bi-Feng Yuan, Yu-Qi Feng

**Affiliations:** 1 College of Chemistry and Molecular Science, Wuhan University, Wuhan 430072, China; jianglc@whu.edu.cn (L.-C.J.); renjiezeng21@whu.edu.cn (R.-J.Z.); 2018202030060@whu.edu.cn (C.Z.); 2 School of Bioengineering and Health, Wuhan Textile University, Wuhan 430200, China; 2415383270@wtu.edu.cn (M.M.); 2415383227@wtu.edu.cn (X.-J.C.); yqfeng@whu.edu.cn (Y.-Q.F.); 3 School of Public Health, Wuhan University, Wuhan 430071, China; sussiegu@whu.edu.cn (S.-Y.G.); t.feng@whu.edu.cn (T.F.); bfyuan@whu.edu.cn (B.-F.Y.); 4 Frontier Science Center for Immunology and Metabolism, Wuhan University, Wuhan 430071, China

**Keywords:** small RNA, RNA modifications, *Arabidopsis thaliana*, mass spectrometry, seed germination

## Abstract

**Background**: Small RNA, defined as RNA molecules of less than 200 nucleotides in length, play pivotal regulatory roles in plant growth, development, and environmental stress responses. However, research on modifications in plant small RNA remains limited. **Methods**: In this study, we developed a liquid chromatography–tandem mass spectrometry (LC-MS/MS) method for the simultaneous detection of 41 RNA modifications, facilitating the systematic qualification and quantification of modifications in plant small RNA. **Results**: We identified a total of nine modifications, among which *N*^6^,*N*^6^-dimethyladenosine (m^6,6^A) is a newly identified modification in plant small RNA. Furthermore, we conducted a quantitative analysis of these modifications in *Arabidopsis thaliana* during the germination process and observed significant dynamic changes in their abundance from 1 to 5 days post-germination. Notably, the trends in the contents of these modifications exhibited a strong correlation with the reported gene expression levels of the relevant modifying enzymes and demodifying enzymes, suggesting that these modifications may play essential roles during seed germination and are tightly regulated by the genes of the corresponding enzymes. **Conclusions**: The discovery of these modifications in plant small RNA, coupled with the dynamic changes in their levels during germination, holds great promise for a further understanding of the physiological functions of small RNA modifications and their associated regulatory mechanisms in plant seed germination.

## 1. Introduction

RNA epigenetic modification is a process of modifying newly synthesized RNA molecules into mature products [1]. To date, more than 170 different RNA modifications have been found in living organisms [2], existing in various types of RNA, including ribosomal RNA (rRNA), transfer RNA (tRNA), messenger RNA (mRNA), and small RNA [3,4,5,6]. RNA modifications play an important regulatory role in the structure and function of RNA [7]. In humans, RNA modifications are strongly associated with the pathogenesis of diseases [8]. The dysregulation of RNA modifications can impair the dynamic tuning of the proteome [9] and disrupt local/global translational rates [10], contributing to the development of various disorders, including cancer, cardiovascular diseases, genetic birth defects, metabolic disorders, and neurological conditions. In plant research, RNA modifications have also been found to play critical roles in biological processes such as reproduction, growth, and development [11]. RNA modifications can be dynamically added and removed by writing and erasing proteins [12,13] and are quantitatively regulated to support normal growth and development processes [14] or to respond to environmental stressors [15,16], providing a new perspective of gene expression regulation and fundamental life processes.

Small RNA is defined as polymeric ribonucleic acid molecules that are less than 200 nucleotides in length and play various essential roles within cells [4]. Small RNA encompasses transfer RNA (tRNA), microRNA (miRNA), PIWI-interacting RNA (piRNA), small nuclear RNA (snRNA), small nucleolar RNA (snoRNA), and tRNA-derived small RNA (tsRNA). In plants, it has been documented that a total of 21 modification types are present within plant small RNA molecules, encompassing 2′-*O*-methyladenosine (Am), 2′-*O*-methylcytidine (Cm), 2′-*O*-methylguanosine (Gm), 2′-*O*-methyluridine (Um), *N*^1^-methyladeosine (m^1^A), 2-methyladeosine (m^2^A), *N*^6^-methyladeosine (m^6^A), *N*^6^-methyl-*N*^6^-threonylcarbamoyladeosine (m^6^t^6^A), *N*^6^-threonylcarbamoyladeosine (t^6^A), 5-methylcytidine (m^5^C), *N*^4^-acetylcytidine (ac^4^C), 1-methylguanosine (m^1^G), *N*^2^-methylguanosine (m^2^G), *N*^2^,*N*^2^-dimethylguanosine (m^2,2^G), *N^7^*-methylguanosine (m^7^G), 5-methyluridine (m^5^U), 5-carbamoylmethyluridine (ncm^5^U), Inosine (I), 1-methylinosine (m^1^I), Dihydrouridine (D), and Pseudouridine (Ψ) [17,18,19].

These modifications are intricately linked to numerous vital agricultural traits. The installation of m^6^A in U6 snRNA by FIONA1 is critical for maintaining downstream mRNA stability, thereby influencing phytochrome signaling and floral transition [20]. Furthermore, 2′-*O*-methylation has emerged as a fundamental requirement for ensuring small RNA activity and metabolism, as its absence in *Arabidopsis thaliana* (*A. thaliana*) results in decreased fertility and the production of shortened fruits [21]. Notably, following the transfer of the animal m^6^A demethylase FTO (Fat Mass and Obesity-associated) into rice and potatoes by Professor Jia Guifang’s research team, the RNA exhibited a substantial reduction in m^6^A levels, specifically, approximately 7% demethylation in poly(A) RNA and a notable 35% decrease in m^6^A within non-ribosomal nuclear RNA. Rice plants grown in greenhouses containing these demethylated RNAs demonstrated a threefold yield increase, while field-grown rice and potatoes also experienced a significant 50% yield enhancement [22]. Consequently, research endeavors into plant RNA modifications hold promise for pioneering novel approaches to enhance agronomic traits and plant breeding. The accurate detection of RNA modifications stands as the cornerstone for functional research in this field. However, in spite of the wide application of RNA detection methods, such as capillary electrophoresis (CE) and LC-MS/MS in recent studies [23,24,25,26], a systematic and comprehensive profiling of modifications in plant small RNA remains elusive.

Seeds constitute the distinctive reproductive organs of plants and are regarded as the “chip” of modern agriculture, wherein their quality exerts a direct influence on crop yield and quality. Seed germination is often considered the beginning of the plant life cycle. Extant research has elucidated the pivotal role of epigenetic modifications in governing seed germination [27]. Nonetheless, the majority of current investigations have primarily concentrated on DNA methylation and histone methylation phenomena [28]. To the best of our knowledge, there has been a notable absence in the literature detailing functional studies on modifications occurring within plant small RNA during seed germination. A comprehensive characterization of small RNA modifications in plants would significantly augment our comprehension of their physiological roles and regulatory mechanisms during seed germination.

**Figure 1:**
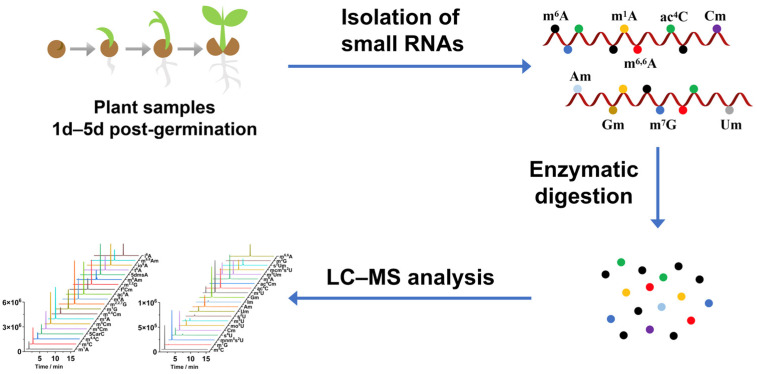
Workflow for profiling modifications in small RNAs of *Arabidopsis thaliana* from 1 to 5 days post-germination using chromatography–mass spectrometry (LC–MS) analysis.

In this study, we developed a liquid chromatography–mass spectrometry (LC-MS) method capable of concurrently detecting 41 distinct RNA modifications, thereby enabling a systematic analysis of the modifications of plant small RNA (Figure 1). Our methodology allowed us to identify nine modifications in the small RNA of *A. thaliana*, including a newly discovered modification, *N*^6,6^-dimethyladenosine (m^6,6^A). Through quantitative analysis spanning the *A. thaliana* germination process, we observed significant dynamic fluctuations in these modifications from 1 to 5 days post-germination. These modifications exhibited correlations with the expression patterns of genes involved in both the addition and removal of these modifications. Our findings contribute to a deeper understanding of the physiological functions of small RNA and their regulatory pathways during plant seed germination.

## 2. Materials and Methods

### 2.1. Chemicals and Reagents

A total of 45 nucleoside standards and an isotopic nucleoside standard (rC-^13^C_5_) were purchased from various commercial sources. The detailed information (CAS numbers, molecular formulae, and molecular weights) of these 45 nucleosides and the isotopic nucleoside can be found in Table S1 in the Supporting Information. CIAP (calf intestinal alkaline phosphatase) and S1 nuclease were obtained from Takara Biotechnology (Dalian, China). Venom phosphodiesterase I was obtained from Sigma-Aldrich (Beijing, China). LC-MS grade methanol (MeOH) was purchased from FTSCI Co., Ltd. (Wuhan, China). Analytical grade formic acid (FA) was purchased from Sinopharm Chemical Reagent Co., Ltd. (Shanghai, China).

### 2.2. Plant Materials

The *Arabidopsis thaliana* (*A. thaliana*, Columbia ecotype), cultured according to a previously reported procedure [29]. The *A. thaliana* was grown in a greenhouse at 23 °C (day/night). The sterilized seeds of *A. thaliana* were sown on Murashige & Skoog medium with 1% sucrose and 0.8% agar. The seeds were then incubated at 4 °C in darkness for 2 days before being transferred to the growth chamber. Plant samples growing under normal conditions for 1 day(d), 2 d, 3 d, 4 d, and 5 d were harvested separately. All harvested plant samples were weighted and stored at −80 °C.

### 2.3. Purification of Small RNAs

The small RNAs of plant samples were extracted using the miRcute Plant miRNA Isolation Kit from Tiangen Biotech (Beijing, China) after being ground in liquid nitrogen. The experimental process was strictly carried out in accordance with the official instructions. The small RNA solution obtained was detected using a B500 UV spectrophotometer (Metash, Shanghai, China) for its concentration, with values of A260/280 and A260/230.

### 2.4. Enzymatic Digestion of Small RNAs

The enzymatic digestion of purified small RNAs was performed according to previously described procedures [24]. Briefly, 2 μL of 10× buffer (500 mmol/L Tris–HCl, 10 mmol/L MgCl_2_, 100 mmol/L NaCl, 10 mmol/L ZnSO_4_, pH 7.0), 0.5 μL of S1 nuclease (180 U/mL), 0.5 μL of venom phosphodiesterase I (0.001 U/mL), and 0.25 μL of CIAP (30 U/mL) were added to 0.08–2 μg of RNA dissolved in H_2_O, reaching a total volume of 20 μL. The mixture was incubated at 37 °C for 6 h. Then, 280 μL of H_2_O and 300 μL of chloroform were added, followed by vortexing for 3 min and centrifugation at 12,000 rpm for 5 min. The upper aqueous phase was collected. The chloroform extraction was repeated three times. The aqueous phase containing nucleosides was collected and lyophilized to dry and then subjected to LC-MS analysis.

### 2.5. LC-MS/MS Analysis

The analysis of nucleosides was performed on a Shimadzu 8050 mass spectrometer (Kyoto, Japan). The digested nucleosides were separated using a Shim-pack GIST C18 column (100 mm × 2.1 mmi.d., 2.0 μm, Shimadzu, Kyoto, Japan) with reverse-phase UHPLC. The column temperature was set at 40 °C. Solvent A of 0.05% FA/H_2_O and solvent B of MeOH were used for the chromatographic separation with a flow rate of 0.3 mL min^−1^. The gradient for the separation was 0−2 min, 95% B; 2–10 min, 95–20% B; 10–12 min, 20% B; 12–12.5 min, 20–95% B; 12.5–15 min, 95% B. Mass spectrometry analysis was operated in positive-ion mode. The resolution was around 0.7 Da. The multiple reaction monitoring (MRM) was utilized for detection. The optimized mass spectrometric parameters are listed in Table S2 in the Supporting Information.

The calibration curves were built to quantitatively measure the nucleosides. Different concentrations of nucleosides, ranging from 1 ppb to 1 ppm, were mixed with a fixed amount of rC-^13^C_5_ (0.1 nmol). The calibration curves were constructed by plotting the peak area ratios (nucleosides/rC-^13^C_5_) against the various amounts of nucleosides. The values of the intercept and slope of the calibration curves are listed in Table S3.

### 2.6. High-Resolution Mass Spectrometry Analysis

The detected modifications were examined using a high-resolution LTQ-Orbitrap Elite mass spectrometer (Thermo-Fisher Scientific, Waltham, MA, USA) equipped with an ESI source and a Dionex Ultimate 3000 UPLC system (Thermo-Fisher Scientific, Waltham, MA, USA). The MS analysis was performed in positive-ion mode with full scan detection (*m*/*z* 110–350) at a resolution of 60,000. Collision-induced dissociation (CID) with a collision energy of 13–17 eV was used. The source and ion transfer parameters applied are as follows: auxiliary gas flow, 15 arbitrary units; sheath gas flow, 35 arbitrary units; capillary temperature, 350 °C; heater temperature, 300 °C; auxiliary gas flow, 15 arbitrary units; sheath gas flow, 35 arbitrary units; capillary voltage, 35 V; spray voltage, 3.5 kV; the S-lens RF level, 60%. Data analysis was achieved using Xcalibur (Thermo Fisher Scientific, Waltham, MA, USA).

### 2.7. Gene Expression Analysis

The analysis of relative gene expression levels was conducted in accordance with previously established protocols [30]. Total RNAs were extracted from *A. thaliana* tissues utilizing the RNAprep Pure PlantPlus Kit (TIANGEN). Subsequently, first-strand complementary DNA (cDNA) was synthesized using the ABScript II cDNA First-Strand Synthesis Kit (ABClonal, Wuhan, China). The cDNA was then amplified by employing the TransStart^®^ Top Green qPCR SuperMix (TransGen, Beijing, China) in a CFX Connect Real-Time PCR Detection System (Bio-Rad, Shanghai, China). ACTIN2 (AT3G18780) served as an internal control. The primer sequences utilized for the qRT-PCR experiments are listed in Table S5.

### 2.8. Statistical Analysis

The experimental data were processed with Origin 2024 software (OriginLab Corporation, Northampton, MA, USA). The *p* value < 0.05 was considered to have statistical significance.

## 3. Results and Discussion

### 3.1. Establishment of LC-MS/MS Analytical Method

To comprehensively screen for modifications in small RNA during seed germination, we first established an LC-MS/MS method for the integrated analysis of modifications in the small RNA of *A. thaliana*. By optimizing chromatography separation conditions and mass spectrometry parameters, we successfully developed an LC-MS/MS method capable of simultaneously detecting 41 modified nucleosides (Table S2 and Figure S1). Among these nucleosides, there are six groups of isomers with similar molecular weights (m^1^A, m^2^A, m^6^A, and m^8^A; m^3^C and m^5^C; m^1^G, m^2^G, and m^7^G; s^2^U and s^4^U; m^3^U and m^5^U; m^4^Cm and m^5^Cm), which share identical MS transitions under multiple reaction monitoring (MRM). However, under the LC-MS/MS conditions employed, these isomers can be separated based on their different retention times (Figure S1), providing critical support for our qualitative and quantitative studies of modifications in small RNA.

### 3.2. Qualitative Analysis of Modifications in Small RNA

Using the established method, we digested small RNA extracted from dry seeds and plant materials of *A. thaliana* at different germination stages into nucleosides and detected the RNA modifications present using LC-MS/MS. The RNA modifications detected in the experiments were confirmed based on the consistency of retention times and MS^2^ mass spectrometry data between the detected modifications and nucleoside standards. According to this criterion, by comparing with forty-one nucleoside standards, a total of nine modifications (Am, Cm, Gm, Um, m^7^G, m^1^A, m^6^A, m^6,6^A, and ac^4^C) were detected in the small RNA of *A. thaliana*.

**Figure 2:**
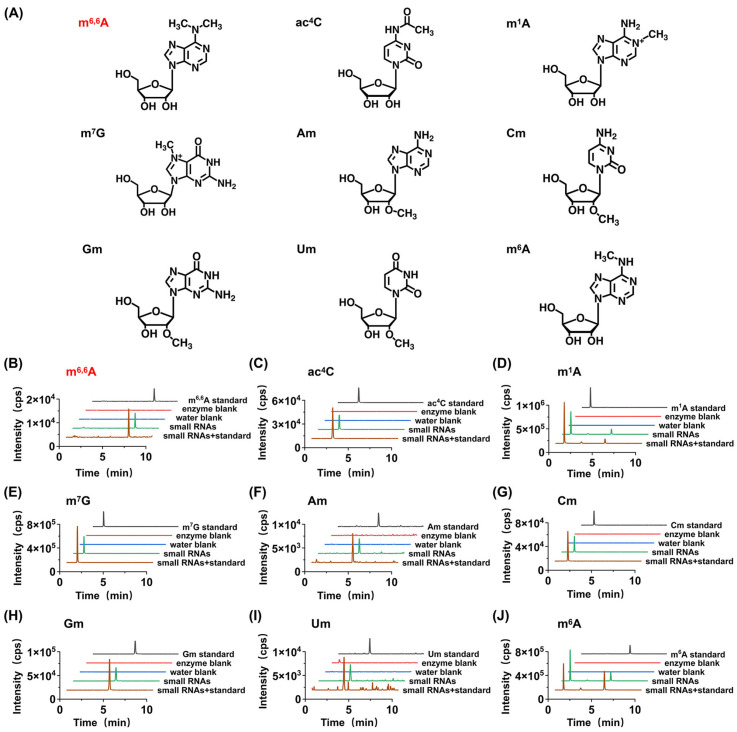
Determination of modifications in the small RNAs of *A. thaliana*. (**A**) The structures of detected modifications. (**B**) The extracted ion chromatograms of the newly detected modifications in m^6,6^A from small RNAs with spiked standards, small RNAs, H_2_O blank, enzyme blank, and nucleoside standards. (**C**–**J**) The extracted ion chromatograms of the modifications in ac^4^C, m^1^A, m^7^G, Am, Cm, Gm, Um, and m^6^A from small RNAs with spiked standards, small RNAs, H_2_O blank, enzyme blank, and nucleoside standards.

To further confirm the modified nucleosides discovered in *A. thaliana* small RNA, we added the standards of the aforementioned modifications to the digested nucleosides of *A. thaliana* small RNA. The results showed that the retention times of the spiked nucleosides were consistent with those in *A. thaliana*. Simultaneously, we examined the contamination of these modifications being present in the RNA digestion enzymes or the water used in the experiment. It was observed that these modifications were not detected in the blank control experiments using RNA enzymes and water, eliminating the possibility that the detected modifications originated from contamination by digestive enzymes or water (Figure 2).

**Figure 3:**
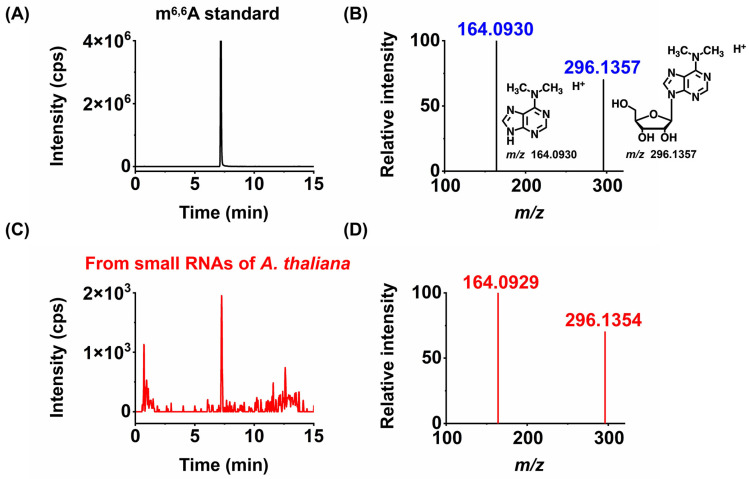
Identification of new modifications in small RNAs of *A. thaliana* by high-resolution mass spectrometry. The extracted ion chromatograms and product ion spectra from the m^6,6^A standard (**A**,**B**) and small RNAs of *A. thaliana* (**C**,**D**).

Notably, among the nine modifications detected in the small RNA of *A. thaliana* (Am, Cm, Gm, Um, m^7^G, m^1^A, m^6^A, m^6,6^A, and ac^4^C), m^6,6^A has not been reported in previous studies in plant small RNA [17,18,19]. Therefore, we further employed high-resolution MS analysis to confirm the newly discovered modification in the small RNA of *A. thaliana*. The results indicated that the modification not only exhibited similar retention times in chromatography (Figure 3) but also had the same mass-to-charge ratios of parent and daughter ions detected by high-resolution MS as their corresponding theoretical values with a mass error of 1 ppm (Figure 3), further confirming the presence of the detected modification. In summary, we identified a newly discovered RNA modification, m^6,6^A, in the small RNA of the dry seeds and plant materials of *A. thaliana* at different germination stages.

### 3.3. Quantitative Analysis of Modifications in Small RNA of A. thaliana at Various Germination Stages

Next, we conducted the quantitative detection of the nine modifications (Am, Cm, Gm, Um, m^7^G, m^1^A, m^6^A, m^6,6^A, and ac^4^C) found in small RNA from the dry seeds and plant materials of *A. thaliana* at different germination stages. Using the internal standard method, various amounts of nucleoside standards and fixed amounts of the isotope internal standard (rC-^13^C_5_) were mixed to construct the calibration curves by plotting the peak area ratios (nucleosides/rC-^13^C_5_) against the amounts of nucleosides. By determining the relationship between the peak area ratio (nucleoside/rC-^13^C_5_) and the amount of nucleoside, we constructed quantitative curves and obtained relevant slope and intercept data (Table S3). The results showed that all plotted quantitative standard curves exhibited high linearity, with determination coefficients (R^2^) higher than 0.99. The limits of detection (LODs) of the detected RNA modifications ranged from 1.3 fmol to 253.1 fmol. We also evaluated the accuracy and precision of the quantitative method by calculating relative errors (REs) and intra-day and inter-day relative standard deviations (RSDs). The results indicated that the REs and RSDs of this method were less than 10.0% and 8.2%, respectively (Table S4), suggesting good accuracy and precision of the quantitative method employed in the experiment.

**Figure 4:**
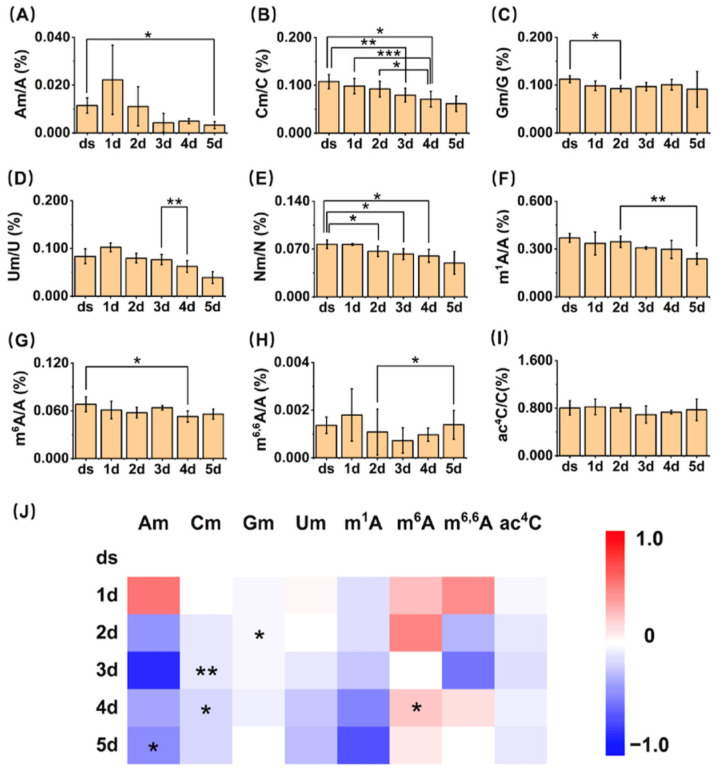
Quantification of modifications in small RNAs of *A. thaliana*. (**A**–**I**) Quantification of Am, Cm, Gm, Um, Nm, m^1^A, m^6^A, m^6,6^A, and ac^4^C in small RNAs of *A. thaliana*. *, *p* < 0.05; **, *p* < 0.01; ***, *p* < 0.001. Error bars represent standard deviation (*n* = 3). (**J**) Heatmap showing the contents of modifications in small RNAs of *A. thaliana*.

Among them, eight modifications (Am, Cm, Gm, Um, m^1^A, m^6^A, m^6,6^A, and ac^4^C) in small RNA from the dry seeds and plant materials of *A. thaliana* at different germination stages could stably reach the limits of quantitation (LOQs). Therefore, based on the plotted standard curves, we successfully analyzed the contents of these eight modifications and calculated the relative quantities of modified nucleosides to their corresponding unmodified nucleosides (Figure 4). The quantification results revealed the following ranges for *A. thaliana* 1 to 5 days post-germination: Am (0.003–0.022%, Am/A), Cm (0.06–0.11%, Cm/C), Gm (0.09–0.11%, Gm/G), Um (0.04–0.10%, Um/U), m^6^A (0.05–0.07%, m^6^A/A), m^1^A (0.24% to 0.37%, m^1^A/A), m^6,6^A (0.0007% to 0.0018%, m^6,6^A/A), and ac^4^C (0.69% to 0.82%, ac^4^C/C). Using dry seeds as the baseline, we quantitatively assessed the dynamic changes in the levels of these eight modifications throughout the seed germination process (from 1 d to 5 d) in *A. thaliana*.

### 3.4. The Contents of 2′-O-Methylation Modification (Nm) Showed a Decreasing Trend During the Germination Process

The methylation modifications of Nm (including Am, Cm, Gm, and Um) all occur at the 2′ position of the ribose of their respective nucleosides. This type of modification is abundant and conserved in organisms, and almost all miRNAs in *A. thaliana* have their 3′-ends fully 2′-O-methylated [19]. In previous studies, the absence of Nm modification led to more 3′ tailing (mainly uridylation) and 3′-to-5′ trimming on miRNAs, reducing their stability, while miRNAs with Nm modification further formed RNA-induced silencing complexes (RISC), cleaving or inhibiting mRNA to reduce corresponding gene expression [31], thereby ensuring normal processes such as plant root and leaf development. The absence of 2′-O-methylation in miRNA leads to delayed growth, morphological defects, and reduced fertility [32]. However, there have been no reports on the dynamic changes in Nm modification during seed germination. Our quantitative results showed that starting from the second day of germination, the contents of Am, Cm, Gm, and Um all exhibited a certain decreasing trend. Am rapidly decreased to 20% of the first day on the third day of germination, with subsequent slight fluctuations, but still a downward trend. Cm gradually decreased to 62% of the first day of germination. Gm showed a smaller change, decreasing to 92% of the first day on the fifth day of germination. Um gradually decreased to 38% of the first day of germination. Due to the structural similarity of these modifications, we also compared the changes in the overall Nm content (sum of Am, Cm, Gm, and Um) during the seed germination process. The results indicated that the Nm content also showed a gradual decreasing trend, decreasing to 65% of the first day of germination on the fifth day. Germination is a highly coordinated process that involves rapid transcriptional and post-transcriptional reprogramming. The expression and activity of RNA modification enzymes (writers) and demodifying enzymes (erasers) are likely dynamically regulated to meet the developmental demands of seed germination. The observed decline in the abundance of these modifications may be attributed to the downregulation of genes encoding modifying enzymes and the upregulation of genes encoding demodifying enzymes.

**Figure 5:**
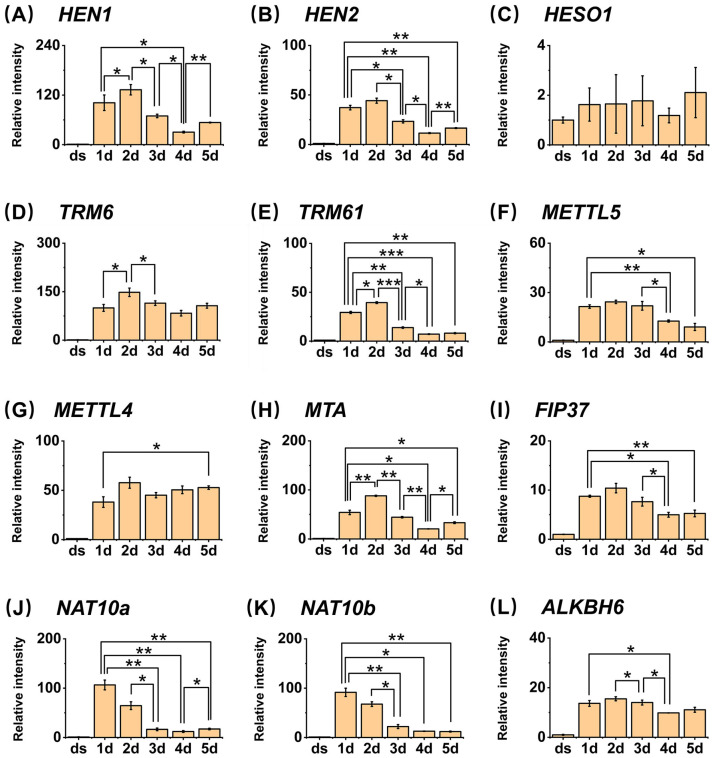
Relative expression levels of *HEN1*, *HEN2*, *HESO1*, *TRM6*, *TRM61*, *METTL5*, *METTL4*, *MTA*, *FIP37*, *NAT10a*, *NAT10b*, and *ALKBH6* of *A. thaliana*. *, *p* < 0.05; **, *p* < 0.01; ***, *p* < 0.001. Error bars represent standard deviation (*n* = 2).

Research on genes responsible for Nm modification on small RNA will help us further understand the complete pathway through which Nm modification functions. The HUA Enhancer 1 (*HEN1*) protein is the first identified methyltransferase responsible for the Nm modification processing of small RNA, first found in *A. thaliana* miRNAs, and the homologs of *HEN1* have also been found in other plants [19]. *A. thaliana* mutants lacking *HEN1* exhibit features of small leaves and short stems [33], further supporting the necessity of Nm modification of small RNA in the normal development of *A. thaliana*. Therefore, we investigated the expression patterns and relative expression levels of these three genes during seed germination (Figure 5). The relative expression levels of the *HEN1* gene during seed germination showed that it exhibited a gradual decreasing trend starting from the second day of germination, which was consistent with the gradual decrease in Nm modification contents over time. The HUA ENHANCER 2 (HEN2) encodes a potential DExH-box RNA helicase functioning redundantly with HEN1. It degrades mis-spliced, noncoding, and prematurely terminated RNAPII transcripts [34]. HEN2 interacts with the core nuclear exosome, the cap-binding complex (which binds to the 5′cap of newly synthesized RNA), and the nuclear exosome targeting complex [35]. The *hen2-2* mutant is sensitive to cold [35]. In our study, the expression level of *HEN2* also gradually decreased starting from the second day of seed germination (Figure 5). *HESO1* is an inhibitor of Nm modification generation, and its expression level was opposite to that of *HEN1* and *HEN2*, gradually increasing starting from the first day of seed germination. Accordingly, we also investigated the expression patterns of these three genes during seed germination stages by referring to the public database BAR (http://bar.utoronto.ca/eplant (accessed on 8 January 2025)). The relative expression levels of each gene observed in our results were in close alignment with the data retrieved from the database (Figure S2). Therefore, these genes may precisely regulate the dynamic changes in Nm modification, thereby precisely regulating the seed germination process.

In past studies on the mechanism of small RNA action, some miRNAs have been shown to promote the development and maintenance of the shoot apical meristem by regulating targets in the STM-WUS-CLV pathway; for example, miR394 can regulate the expression level of the WUS protein by affecting the expression of the LCR protein [36]. This process may also be regulated by Nm modification, and fluctuations in Nm content may regulate the STM-WUS-CLV pathway by modulating the stability of related miRNAs. The specific mechanisms and targeted mRNAs of Nm modification during seed germination remain to be further explored.

### 3.5. The Contents of Methylation Modifications (m^1^A, m^6^A, and m^6,6^A) Also Exhibited a Decreasing Trend During the Germination Process

m^1^A, m^6^A, and m^6,6^A represent methylation modifications occurring at corresponding positions on adenosine bases. m^6^A modification received tremendous attention in past studies and is also found to exist in small RNA in our results. Regarding the mechanism of dynamic regulation, m^6^A in plants is often catalyzed by S-adenosylmethionine (SAM) in conjunction with proteins such as MTA, MTB, and FIP37. These methyltransferases, along with demethylases ALKBHs, collectively contribute to the dynamic regulation of m^6^A [37]. Experiments using *MTA* mutants to reduce the m^6^A content of pri-miRNA indeed resulted in decreased miRNA synthesis. Further experimental results indicate that reduced m^6^A content in *A. thaliana* miRNAs leads to decreased auxin signaling, affecting root and stem elongation and development [17].

m^1^A has been reported in tRNA, rRNA, and mRNA [38], with higher abundance in tRNA and rRNA, but its specific sites and functions in mRNA remain unclear. In this study, it has been detected in small RNA in plants. To obtain more information about details on how m^1^A is adjusted, we noticed that *TRM6* and *TRM61* have been identified as the tRNA m^1^A methyltransferase in *A. thaliana* [39]. Under enzyme regulation, RNAs carrying m^1^A modifications help form the correct structure of tRNA, thereby regulating life activities and ensuring the normal development of structures such as leaves [18].

m^6,6^A is a newly discovered modification type in small RNA from Arabidopsis seed material in this study [40], which ranged from 0.0007% to 0.0018% (m^6,6^A/A). Current research on m^6,6^A mainly focuses on the demethylation of two consecutive adenosines in the conserved stem–loop structure, helix45, at the 3′ end of rRNA. In studies of related sites in animals, the m^6,6^A modification at this site is catalyzed by the TFB1M methyltransferase and can be regarded as a marker for the normal assembly of mitochondrial ribosomal subunits in eukaryotes [41]. However, the corresponding sites and functions of m^6,6^A modifications in other types of RNA or in plants are still unclear.

In the quantitative results of our experiments, the overall trends in the changes in the two methylation modifications, m^1^A and m^6^A, demonstrate a gradual decline. Specifically, the peak of m^6,6^A modification occurs on the first day of germination, followed by a decline on the second day. Additionally, we analyzed the gene expression levels of methyltransferases and demethylases responsible for m^1^A and m^6^A (Figure 5). We found that the trends in *MTA* and *FIP37*, which assist in catalyzing the production of m^6^A, are basically consistent with the trends in m^6^A content. The expression of *MTA* exhibits a marked increase on the first and second days of germination, followed by a decreasing trend from the third day onward. Analogously, the expression of *FIP37* significantly elevates during the initial two days of germination and subsequently decreases on the third day. The patterns of gene expression alterations for TRM61 and TRM6 exhibit a striking similarity to the trends observed for *MTA* and *FIP37*. Specifically, there is an initial increase in expression during the first two days of germination, followed by a subsequent decline starting from the third day. These expression changes correspond precisely with the fluctuations observed in m^1^A content, suggesting a potential regulatory link between these genes and m^1^A modification during the germination process. Similarly, the demethylase ALKBH6 demonstrates a pattern of initial increase during the first two days post-germination, followed by a decrease from the third to the fifth day post-germination. Notably, the relative expression levels of each of these genes in our results are also in close concordance with the data retrieved from the BAR database, as illustrated in Figure S2. These results suggest that these genes may also participate in the production and demethylation of m^6^A on small RNA, and these methylation modifications may share similar regulatory mechanisms in metabolic processes within organisms. Furthermore, given the remarkable similarity between the variation patterns of these gene expressions and the content of m^6,6^A modification, they may also participate in the methylation and demethylation of m^6,6^A.

### 3.6. The Content of ac^4^C Gradually Decreases During Germination

ac^4^C, an acetylation modification occurring at the *N*^4^ position of cytosine, has received widespread attention as the only known acetylation modification on RNA. Previous studies have identified ac^4^C modification on noncoding RNA in plants, and in the past two years, it has also been found in the mRNAs of *A. thaliana* [42]. Our results also prove the existence of ac^4^C in small RNA. Compared with our results of the content of ac^4^C in small RNA, the overall content of ac^4^C in *A. thaliana* RNA is 0.115% ± 0.005% (compared to unmodified cytosine) in previous studies [42]. Past studies have shown that ac^4^C in RNA can promote the formation of growth and development-related small RNA by extending the lifespan of the miRNA biosynthesis factor TOUGH (TGH), thereby ensuring the normal growth and development of the plant [43].

In the quantitative analysis, the content of ac^4^C undergoes a gradual decline during the germination process of *A. thaliana*. This observation implies a potential role for ac^4^C modification in influencing seed germination, potentially through its modulation of small RNA. Nonetheless, to date, enzymes responsible for catalyzing both the formation and removal of ac^4^C modification remain unidentified in plants. Consequently, a more thorough investigation is warranted to elucidate the specific mechanisms underlying its function.

## 4. Conclusions

In summary, following the establishment of an LC-MS/MS method for the simultaneous detection of 41 modifications, we undertook qualitative and quantitative analyses of small RNA modification types in *A. thaliana* seed materials spanning from 1 to 5 days of germination. Qualitative results showed that we identified a total of nine modifications in small RNA from *A. thaliana* seed materials, including Am, Cm, Gm, Um, m^7^G, m^1^A, m^6^A, m^6,6^A, and ac^4^C. Subsequently, our quantitative results demonstrated that the abundances of Nm modifications (comprising Am, Cm, Gm, and Um), methylation modifications (m^1^A and m^6^A), and ac^4^C exhibited a gradual decline, commencing from the second day of germination. Specifically, the content of m^6,6^A initially increased on the first day of germination but subsequently decreased. Additionally, we conducted an analysis of the gene expression levels of the enzymes known to be involved in the modification and demodification processes, finding them to be highly congruent with the observed changes in the modification content. Future research endeavors focusing on the enzymes associated with these modifications will contribute to a deeper understanding of the physiological roles and regulatory mechanisms of these modifications in plant seed germination processes.

## Data Availability

The original contributions presented in this study are included in the article and Supplementary Material. Further inquiries can be directed to the corresponding authors.

## Supplementary Materials

The following supporting information can be downloaded at: https://www.mdpi.com/article/10.3390/metabo15050319/s1, Table S1: The information of 45 nucleosides standards and one isotopic nucleoside standard; Table S2: The MRM parameters for analysis of nucleosides by LC-MS using a Shimadzu 8050 mass spectrometer; Table S3: Calibration curves, LODs and LOQs for the analysis of nucleosides by LC-MS; Table S4: Accuracy and precision for the analysis of nucleosides by LC-MS; Table S5: Sequences of PCR primers; Figure S1: Representative extracted-ion chromategrams of 41 modifications by LC-MS analysis with MRM detection modes; Figure S2: Relative expression levels of related genes of A. thaliana in previous studies.
