# Supplementary material for: Comprehensive Analysis of Small RNA Modifications in Arabidopsis thaliana and Their Dynamics During Seed Germination
Source: Metabolites. 2025 May 10;15(5):319. doi: 10.3390/metabo15050319 (PMC12113266; doi:10.3390/metabo15050319)
Supplement: Supplementary file 1 [file metabolites-15-00319-s001.zip › metabolites-3601466-supplementary.pdf]

Supplementary Materials

Comprehensive analysis of small RNA modifications in *Arabidopsis thaliana* and their dynamics during seed germination

Liu-Cheng Jiang[1], Meng Men[2], Xuan-Jun Cui[2], Ren-Jie Zeng[1], Shu-Yi Gu[3], Tian Feng[3], Chen Zeng[1], Tian-Tian Ye[1,2,*], Jun Xiong[3,*], Bi-Feng Yuan[3], Yu-Qi Feng[2,3,4]

[1] College of Chemistry and Molecular Science, Wuhan University, Wuhan, Hubei, 430072, P. R. China; jianglc@whu.edu.cn (L.-C.J.); renjiezeng21@whu.edu.cn (R.-J.Z.); 2018202030060@whu.edu.cn (C.Z.)

[2] School of Bioengineering and Health, Wuhan Textile University, Wuhan 430200, China; 2415383270@wtu.edu.cn (M.M.); 2415383227@wtu.edu.cn (X.-J.C.); yqfeng@whu.edu.cn (Y.-Q.F.)

[3] School of Public Health, Wuhan University, Wuhan, Hubei, 430071, P. R. China; sussiegu@whu.edu.cn (S.-Y.G.); T.Feng@whu.edu.cn (T.F.); (B.-F.Y.)

[4] Frontier Science Center for Immunology and Metabolism, Wuhan University, Wuhan 430071, China

* Correspondence: ye_tiantian@whu.edu.cn (T.T.Y.); jxiong@whu.edu.cn (J.X.)

List of tables and figures

Table S1. The information of 45 nucleosides standards and one isotopic nucleoside standard.

Table S2. The MRM parameters for analysis of nucleosides by LC-MS using a Shimadzu 8050 mass spectrometer.

Table S3. Calibration curves, LODs and LOQs for the analysis of nucleosides by LC-MS.

Table S4. Accuracy and precision for the analysis of nucleosides by LC-MS.

Table S5. Sequences of PCR primers.

Figure S1. Representative extracted-ion chromategrams of 41 modifications by LC-MS analysis with MRM detection modes.

Figure S2. Relative expression levels of related genes of A. thaliana in previous studies.

Table S1. The information of 45 nucleoside standards and one isotopic nucleoside standard.

No	Nucleosides	Abbreviation	CAS number	Molecular formula	Molecular weight	Company
1	adenosine	A	58-61-7	$C_{10}H_{13}N_5O_4$	267.24	Sigma-Aldrich
2	cytidine	C	65-46-3	$C_9H_{13}N_3O_5$	243.22	Sigma-Aldrich
3	guanosine	G	118-00-3	$C_{10}H_{13}N_5O_5$	283.24	Sigma-Aldrich
4	uridine	U	58-96-8	$C_9H_{12}N_2O_6$	244.20	Sigma-Aldrich
5	Cytidine-$^{13}C_5$	rC-$^{13}C_5$	-	$C_4{}^{13}C_5H_{13}N_3O_5$	248.18	CATO
6	2′-O-methyladenosine	Am	2140-79-6	$C_{11}H_{15}N_5O_4$	281.27	J&K Scientific Ltd.
7	N^1-methyladenosine	m^1A	15763-06-1	$C_{11}H_{15}N_5O_4$	281.27	J&K Scientific Ltd.
8	2-methyladenosine	m^2A	16526-56-0	$C_{11}H_{15}N_5O_4$	281.27	Carbosynth
9	N^6-methyladenosine	m^6A	1867-73-8	$C_{11}H_{15}N_5O_4$	281.27	Hanhong Chemical
10	8-methyladenosine	m^8A	56973-12-7	$C_{11}H_{15}N_5O_4$	281.27	Carbosynth
11	N^6,2′-O-dimethyladenosine	m^6Am	57817-83-1	$C_{12}H_{17}N_5O_4$	295.29	Granlen
12	N^6,N^6-dimethyladenosine	$m^{6,6}A$	2620-62-4	$C_{12}H_{17}N_5O_4$	295.29	J&K Scientific Ltd.
13	N^6,N^6,2′-O-trimethyladenosine	$m^{6,6}Am$	30891-53-3	$C_{13}H_{19}N_5O_4$	309.32	Granlen
14	N^6-acetyladenosine	ac^6A	3768-18-1	$C_{11}H_{15}N_3O_6$	285.25	Carbosynth
15	N^6-isopentenyladenosine	i^6A	7724-76-7	$C_{15}H_{21}N_5O_4$	335.36	J&K Scientific Ltd.
16	N^6-(cis-hydroxyisopentenyl) adenosine	io^6A	6025-53-2	$C_{15}H_{21}N_5O_5$	351.36	Carbosynth
17	N^6-threonyl-carbamoyladenosine	t^6A	24719-82-2	$C_{15}H_{20}N_6O_8$	412.36	J&K Scientific Ltd.
18	5′-deoxy-5′-methylthioadensine	5d5msA	2457-80-9	$C_{11}H_{15}N_5O_3S$	297.33	Santa Cruz Biotechnology
19	2′-O-methylcytidine	Cm	2140-72-9	$C_{10}H_{15}N_3O_5$	257.24	Carbosynth

20	*N*[3]-methylcytidine	m^3C	2140-64-9	$C_{10}H_{15}M_3O_5$	257.24	Carbosynth
21	5-methylcytidine	m^5C	2140-61-6	$C_{10}H_{15}N_3O_5$	257.24	Sigma-Aldrich
22	N^4,N^4-dimethylcytidine	$m^{4,4}C$	13007-43-7	$C_{11}H_{17}N_3O_5$	271.27	Carbosynth
23	N^4,N^4,2'-O-trimethylcytidine	$m^{4,4}Cm$	34218-81-0	$C_{12}H_{19}N_3O_5$	285.30	Carbosynth
24	N^4,2'-O-dimethylcytidine	m^4Cm	13048-95-8	$C_{11}H_{15}N_3O_5$	271.27	Carbosynth
25	5,2′-O-dimethylcytidine	m^5Cm	113886-70-7	$C_{11}H_{17}N_3O_5$	271.27	Carbosynth
26	5-formyl-2′-O-methylcytidine	f^5Cm	176858-79-0	$C_{11}H_{15}N_3O_6$	285.26	Granlen
27	5-carboxycytidine	ca^5C	64623-37-6	$C_{10}H_{13}N_3O_7$	287.23	Berry& Associates
28	N^4-acetylcytidine	ac^4C	16265-37-5	$C_{12}H_{15}N_5O_5$	309.28	Carbosynth
29	N^4-acetyl-2′-O-methylcytidine	ac^4Cm	113886-71-8	$C_{12}H_{17}N_3O_6$	299.29	Carbosynth
30	2′-O-methylguanosine	Gm	2140-71-8	$C_{11}H_{15}N_5O_5$	297.27	Carbosynth
31	1-methylguanosine	m^1G	2140-65-0	$C_{11}H_{15}N_5O_5$	297.27	Carbosynth
32	N^2-methylguanosine	m^2G	2140-77-4	$C_{11}H_{15}N_5O_5$	297.27	Carbosynth
33	N^7-methylguanosine	m^7G	20244-86-4	$C_{11}H_{15}N_5O_5$	297.27	Carbosynth
34	N^2,N^2-dimethylguanosine	$m^{2,2}G$	2140-67-2	$C_{12}H_{17}N_5O_5$	311.30	Carbosynth
35	N^2,N^2,7-trimethylguanosine	$m^{2,2,7}G$	40027-70-1	$C_{13}H_{19}N_5O_5$	325.32	Carbosynth
36	2′-O-methyluridine	Um	2140-76-3	$C_{10}H_{14}N_2O_6$	258.23	Carbosynth
37	3-methyluridine	m^3U	2140-69-4	$C_{10}H_{14}N_2O_6$	258.23	Carbosynth
38	5-methyluridine	m^5U	1463-10-1	$C_{10}H_{14}N_2O_6$	258.23	Carbosynth
39	2-thiouridine	s^2U	20235-78-3	$C_9H_{12}N_2O_5S$	260.27	Carbosynth
40	4-thiouridine	s^4U	13957-31-8	$C_9H_{12}N_2O_5S$	260.27	J&K Scientific Ltd.
41	5-methoxyuridine	mo^5U	35542-01-9	$C_{10}H_{14}N_2O_7$	274.23	Carbosynth
42	5,2′-O-dimethyluridine	m^5Um	55486-09-4	$C_{11}H_{16}N_2O_6$	272.26	Carbosynth
43	2-thio-2′-O-methyluridine	s^2Um	113886-72-9	$C_{10}H_{14}N_2O_5S$	274.30	Granlen

44	5-methoxycarbonylmethyl-2-thiouridine	mcm^5s^2U	20299-15-4	$C_{12}H_{16}N_2O_7S$	332.33	Granlen
45	5-methylaminomethyl-2-thiouridine	mnm^5s^2U	32860-54-1	$C_{11}H_{17}N_3O_5S$	303.33	Granlen
46	2′-O-methylinosine	Im	3881-21-8	$C_{11}H_{14}N_4O_5$	282.26	Carbosynth

Table S2. The MRM parameters for analysis of nucleosides by LC-MS using a Shimadzu 8050 mass spectrometer.

Nucleosides	Parent ion(m/z)	Daughter ion(m/z)	Q1(V)	CE(V)	Q3(V)	RT(min)
A	268.10	136.10	-13	-18	-13	4.75
C	244.10	112.10	-20	-13	-19	1.94
G	284.10	152.00	-10	-17	-14	4.93
U	245.10	113.00	-12	-18	-17	3.08
rC-$^{13}C_5$	249.20	112.10	-11	-13	-22	1.96
Am	282.12	136.06	-10	-16	-13	4.09
m^{1}A	282.12	150.08	-10	-21	-14	1.09
m^{2}A	282.12	150.08	-10	-20	-14	2.38
m^{6}A	282.12	150.08	-10	-20	-14	5.56
m^{8}A	282.12	150.08	-11	-21	-14	5.28
m^{6}Am	296.14	150.08	-16	-20	-20	6.62
m^{6,6}A	296.14	164.09	-11	-22	-16	7.15
m^{6,6}Am	310.15	164.09	-20	-20	-20	8.04
ac^{6}A	310.11	178.07	-11	-13	-17	5.54
i^{6}A	336.17	204.12	-12	-18	-20	9.78
io^{6}A	352.16	220.12	-13	-17	-14	7.93
t^{6}A	413.14	282.10	-10	-15	-18	7.26
5d5msA	298.10	136.06	-11	-20	-13	6.67
Cm	258.11	112.05	-10	-13	-10	1.68
m^{3}C	258.11	126.07	-10	-14	-12	0.85
m^{5}C	258.11	126.07	-10	-14	-12	1.26
m^{4,4}C	272.12	140.08	-10	-20	-13	1.40
m^{4,4}Cm	286.14	140.08	-11	-17	-13	2.91

m^4Cm	272.12	126.07	-10	-13	-25	1.58
m^5Cm	272.12	126.07	-10	-13	-12	1.80
f^5Cm	286.10	140.05	-11	-13	-12	5.80
5carC	288.08	156.04	-11	-12	-15	1.17
ac^4C	286.10	154.06	-11	-11	-15	4.86
ac^4Cm	300.11	154.07	-16	-17	-25	4.92
Gm	298.11	152.06	-11	-14	-15	4.34
m^1G	298.11	166.07	-11	-16	-16	3.71
m^2G	298.11	166.07	-19	-18	-30	7.15
m^7G	298.11	166.07	-11	-17	-29	1.01
$m^{2,2}G$	312.13	180.09	-12	-16	-18	5.97
$m^{2,2,7}G$	326.15	194.10	-20	-25	-20	3.35
Um	259.09	113.03	-10	-11	-22	3.09
m^3U	259.09	127.05	-10	-13	-12	0.85
m^5U	259.09	127.05	-10	-13	-12	3.37
s^2U	261.05	129.01	-10	-10	-25	3.79
s^4U	261.05	129.01	-10	-11	-12	1.21
mo^5U	275.10	143.00	-20	-11	-14	3.45
m^5Um	273.11	127.05	-10	-14	-12	1.80
s^2Um	275.07	129.01	-10	-10	-12	6.77
mcm^5s^2U	333.08	201.03	-13	-11	-20	6.44
mnm^5s^2U	304.10	172.05	-11	-12	-17	0.98
Im	283.10	137.05	-11	-13	-12	5.14

Table S3. Calibration curves, LODs and LOQs for the analysis of nucleosides by LC-MS.

Analytes	Calibration curve data			LOD (fmol)	LOQ (fmol)
	Slope	Intercert / 10^{-4}	R^2 value		
A	0.0620	57.8236	0.9918	-	-
C	0.0285	4.8279	0.9975	-	-
G	0.0362	15.5322	0.9961	-	-
U	0.0025	1.3765	0.9919	-	-
Am	1.5379	2.1735	0.9991	4.8	16.2
Cm	0.6896	4.0324	0.9996	1.3	4.4
Gm	0.5586	7.9805	0.9995	5.6	18.7
Um	0.0537	0.9500	0.9978	29.0	96.8
m^1A	1.5747	0.2170	0.9987	3.6	11.9
m^6A	0.6913	1.5066	0.9964	3.9	13.0
$m^{6,6}A$	2.0109	0.1980	0.9965	2.6	8.5
ac^4C	0.0333	0.4820	0.9955	17.5	58.4

Table S4. Accuracy and precision for the analysis of nucleosides by LC-MS.

Analytes	Theoretical value (pmol)	Measured value (pmol)	Relative error (%)	Intra-day (RSD%, n=3)	Inter-day (RSD%, n=3)
	20.00	18.036	-9.8	1.0	3.8
A	200.00	203.821	1.9	2.3	4.1
	2000.00	1993.094	-0.3	5.7	1.7
	20.00	19.120	-4.4	4.8	5.3
C	200.00	201.309	0.7	4.1	0.9
	2000.00	2005.109	0.3	1.2	3.2
	20.00	19.218	-3.9	1.9	4.3
G	200.00	197.004	-1.5	3.7	2.4
	2000.00	2006.110	0.3	1.1	2.0
	20.00	21.523	7.6	5.3	1.1
U	200.00	200.830	0.4	4.1	2.0
	2000.00	2002.997	0.2	4.7	2.0
	0.01	0.0102	2.0	4.6	1.4
Am	0.10	0.102	2.0	7.0	6.5
	1.00	1.030	3.0	3.0	1.7
	0.01	0.0103	3.0	3.0	5.0
Cm	0.10	0.107	7.0	2.1	6.7
	1.00	1.029	2.9	7.2	1.8
	0.01	0.0101	1.0	4.5	7.2
Gm	0.10	0.106	6.0	5.9	6.9
	1.00	1.047	4.7	1.9	6.2
Um	0.01	0.011	10.0	6.3	7.4
Um	0.10	0.102	2.0	6.4	4.0

	1.00	0.984	-1.6	3.7	7.5
	0.01	0.0104	4.0	2.0	3.5
m^1A	0.10	0.103	3.0	1.6	5.9
	1.00	1.047	4.7	4.1	3.2
	0.01	0.0102	2.0	3.5	1.5
m^6A	0.10	0.102	2.0	4.0	3.2
	1.00	1.094	9.4	4.1	7.3
	0.01	0.0101	1.0	2.7	4.2
$m^{6,6}A$	0.10	0.093	-7.0	2.8	6.5
	1.00	1.030	3.0	3.0	8.2
	0.01	0.0102	2.0	6.3	7.0
ac^4C	0.10	0.096	4.0	3.6	3.9
	1.00	0.991	-1.0	7.9	6.3

Table S5. Primer sequences used for qRT-PCR analyses.

Genes	Sequence (from 5′ to 3′)
HEN1	F: 5′-TCCGCTGAACTCTTTAGTGTGT-3'<br>R: 5′-ATCGCCTTTGGAGTAGGAGT-3'
HEN2	F:5′-TGAACTGATGTTGCCACTGC-3'<br>R: 5′-AAACCCATAGCAAAGGTTTCTG-3'
HESO1	F: 5′-GTTGTTCCTTTGGAGTAGTAAAGGG-3'<br>R: 5′-CTCGTATCCCTATCTGCTCGTG-3'
TRM61	F: 5′-GCTGTCTCTGCAAGGGAAGA-3'<br>R: 5′-AGAAGGAACCGCAAGCCAAG-3'
TRM6	F: 5′-TCTGGGTATTCGTCTTGGTGC-3'<br>R: 5′-ACAGCCTCCATTTCTACCATCAAA-3'
METTL5	F: 5′-CAGAACTAGACCGCCACGAT-3'<br>R: 5′-CCAAGCTGCCACCATGAATC-3'
METTL4	F: 5′-GAAGGAGCTCTCGTGGCTTT-3'<br>R: 5′-TGTACCATCCGGTTTCACCTTT-3'
MTA	F: 5′-AGGATTTGAAAGATGTTGAGGCT-3'<br>R: 5′-TCCTCCTTTGCTTTTAAACTTAGCA-3'
FIP37	F: 5′-CCACCAGGGCTTCAGGTAAT-3'<br>R: 5′-TTCTAACACCAGGCGCTACC-3'
NAT10a	F: 5′-CACCATCCTCAGGTTAGGCA-3'<br>R: 5′-GGTCACGAGCCTTATCACCA-3'

NAT10b	F: 5′-TGATCGACGACCTGAGAAGC-3'<br>R: 5′-CCAGTCACAGCACTTGGGAT-3'
ALKBH6	F: 5′-AAGATTACAGAACTGGGGTGGT-3'<br>R: 5′-TTATCTTTGTCAGCCAGGGAGG-3'

(A)

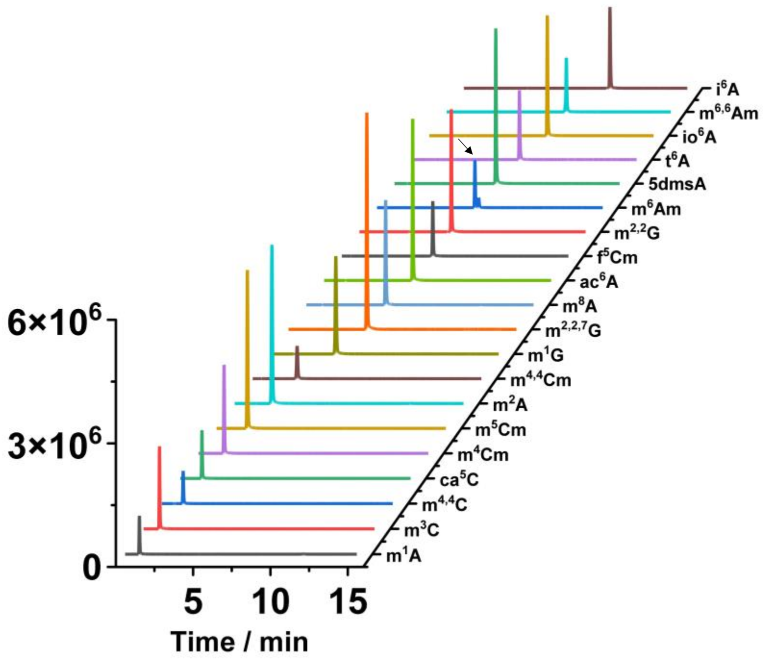

(B)

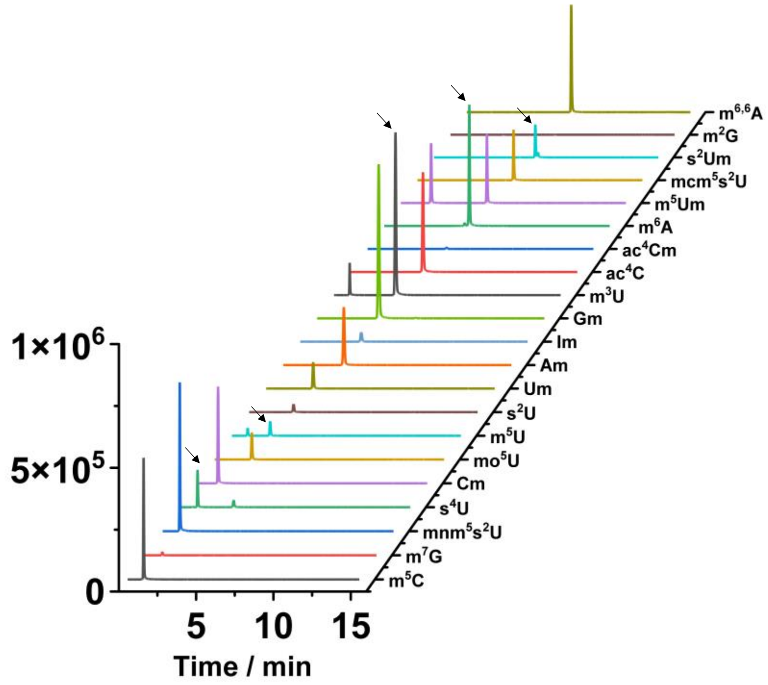

Figure S1. Representative extracted-ion chromatograms of 41 modifications by LC-MS analysis with MRM detection modes. (A) Extracted-ion chromatograms of modifications whose intensity ranges from $1*10^6$ to $6*10^6$ cps. (B) Extracted-ion chromatograms of modifications whose intensity is lower than $1*10^6$ cps.

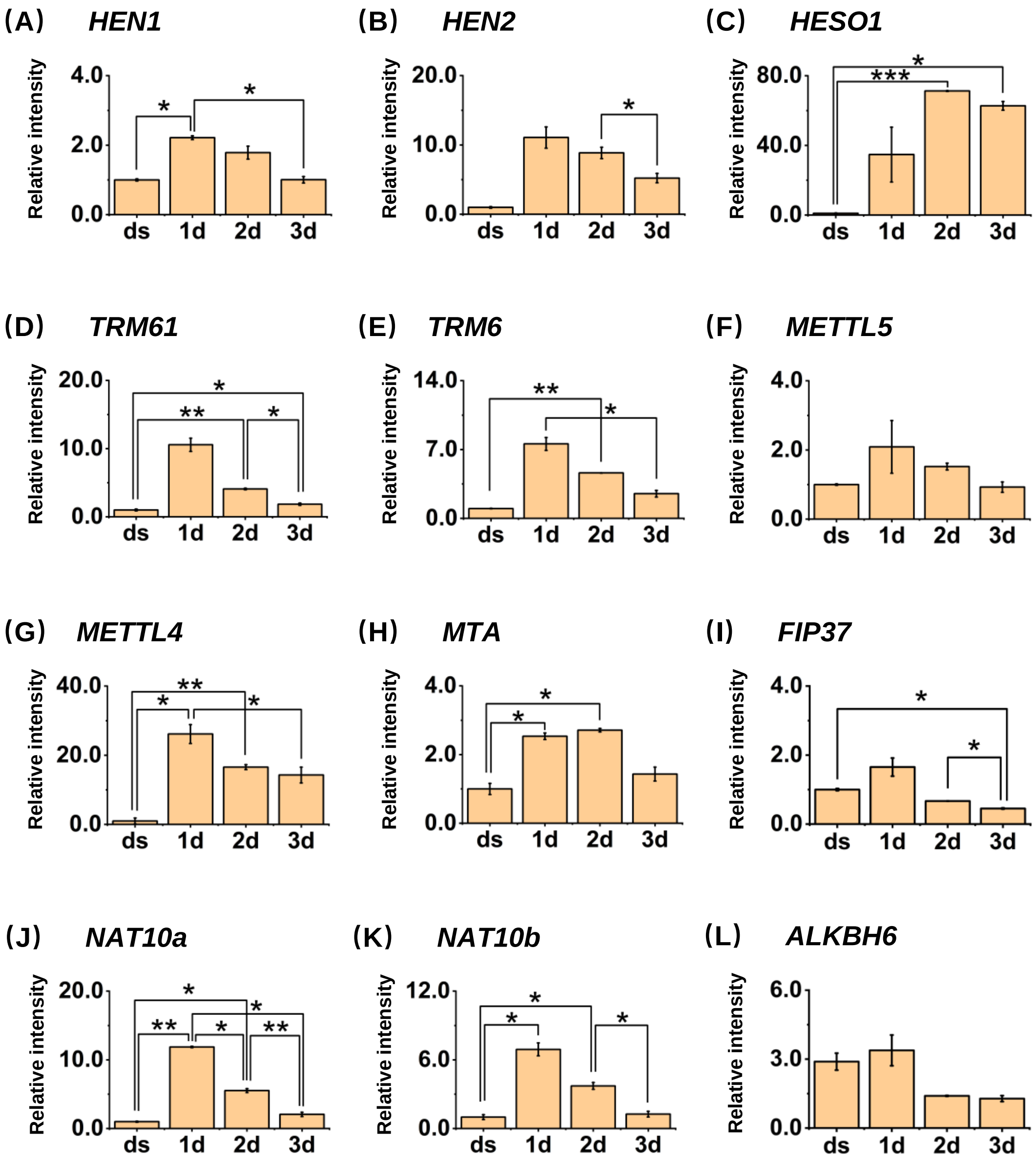

Figure S2. Relative expression levels of related genes of *A. thaliana* in previous studies. Relative expression of *HEN1*, *HEN2*, *HESO1*, *TRM61*, *TRM6*, *METTL5*, *METTL4*, *MTA*, *FIP37*, *NAT10a*, *NAT10b* and *ALKBH6* of *A. thaliana* was annotated based on the data downloaded from the public database BAR (http://bar.utoronto.ca). *, p<0.05; **, p<0.01; ***, p<0.001. Error bars represent standard deviation (n=2).
